# Incipient chronic traumatic encephalopathy in active American football players: neuropsychological assessment and brain perfusion measures

**DOI:** 10.1007/s10072-022-06212-7

**Published:** 2022-06-24

**Authors:** Giacomo Querzola, Carlo Lovati, Maria M. Laganà, Alice Pirastru, Francesca Baglio, Leonardo Pantoni

**Affiliations:** 1 ‘Luigi Sacco’ Department of Biomedical and Clinical Sciences, University of Milan, Via Giovanni Battista Grassi, 74, 20157 Milan, Italy; 2 Headache Center, Neurology Unit, Luigi Sacco Hospital, Milan, Italy; 3 IRCCS, Fondazione Don Carlo Gnocchi ONLUS, Milan, Italy

**Keywords:** CTE, Chronic traumatic encephalopathy, TBI, traumatic brain injury, MRI, NPS, American football

## Abstract

**Background and aims:**

Chronic traumatic encephalopathy (CTE) is a degenerative disease caused by repetitive traumatic brain injury (TBI). Because CTE can be definitely diagnosed only post-mortem, it would be important to explore clinical and radiological correlates of CTE and TBI. The aims of this study were to assess (1) the relationship between the neuropsychological profile of active American football players and the traumatic load; (2) whether traumatic brain injury associated with American football activity has a specific cerebral perfusion pattern; and (3) whether this perfusion pattern correlates with neuropsychological performances.

**Methods:**

In 20 American football players [median age [25th–75th percentile] 25.0 [21.6–31.2] years, all males], we evaluated history, traumatic load and symptoms using the TraQ (Trauma Questionnaire), and cognitive performances on neuropsychological tests. Brain perfusion was estimated using arterial spin labeling MRI and compared to a group of 19 male age-matched (28.0 [24.8–32.3] years) healthy subjects.

**Results:**

We found different cognitive performances between American football players stratified according to field position and career length. Linemen had poorer executive, verbal, and visual performances; a career > 7 years was associated with poorer verbal fluency performances. American football players had statistically significant reduced cerebral blood flow values in sensory-motor areas in comparison with healthy controls. Poorer neuropsychological performances correlated with lower perfusion in specific brain areas.

**Conclusions:**

Our study seems to confirm that CTE in American football players is influenced by the field position and the career length, and correlates with lower cognitive performances linked to lower perfusion in specific brain areas.

**Supplementary Information:**

The online version contains supplementary material available at 10.1007/s10072-022-06212-7.

## Introduction

Chronic traumatic encephalopathy (CTE) is a degenerative disease caused by exposure to repetitive head impacts. It can be seen in contact or collision sport athletes or in military veterans [1, 2]. Neuropathologically, it is characterized by the deposition of paired helical filament tau aggregates in neurons, astrocytes, and cell processes around small blood vessels at the depths of cortical sulci, initially in the frontal, temporal, and parietal cortices and, in later stages, more extensively in the brain and brain stem. This pattern differentiates CTE from other neurodegenerative diseases; for example, unlike Alzheimer’s disease, CTE typically involves neuritic amyloid-beta plaque deposition only in advanced stages of disease. It is unclear if cognitive impairment, mood disorders, and behavioral dyscontrol, frequently observed in patients with CTE, are associated with focal tau deposition, tau-related degeneration, or other consequences of traumatic brain injury (TBI) [1]. It has been observed that dementia development is mainly associated with lesions, such as white matter (WM) rarefaction and dorsolateral frontal cortex neurofibrillary tangles deposition that are related to the length of the career [3].

Previous research has shown conflicting results on the causal role that sports play in the development of CTE. The lack of a causal interpretation hinders a valid surveillance of such activities and prevents clinical, neuropsychological, and neuroimaging comparisons [4–8]. A possible cause of the discrepancy between the results of the studies on CTE is the selection of control groups. Some studies, in fact, do not separate sportsmen from not athletes; others distinguish only “athletes” versus “not athletes,” without considering other characteristics that may create large differences across athletes, such as the type of sport. In some sports, there might also be differences in trauma load according to specific sport characteristics such as the field position. This is typically the case of American football. To reduce these biases, it is necessary to consider the interindividual variability of the sports history and the annual and total trauma load of each athlete. Concerning American football, significant pieces of information are the number of years of activity, the number of games and training sessions per year, the field position, age, and other practiced sports.

Because CTE can be definitely diagnosed only through post-mortem examination, it would be important to have possible clinical and radiological correlates of it. In vivo detection of the disease can help in assessing its epidemiology, risk factors, and course, and finding treatments and prevention strategies. In particular, it is hard to in vivo identify mild traumatic brain injury (mTBI) because its clinical features are usually absent or mild in the acute phase, and the latency between TBIs and CTE development may be of many years [9–12].

Neuropsychological and neuroimaging assessments can be ways for evaluating in vivo CTE. A few neuropsychological and neuroimaging studies on CTE may be found in the literature. TBI has been found related to alterations of cerebral perfusion, evaluated with arterial spin labeling (ASL) techniques, in particular, in American football players. A reduction of thalamic perfusion was observed, and it was associated with cognitive dysfunctions in information processing and speed of response, memory, learning, verbal fluency, and executive functions [13]. Mild but repetitive TBIs have been found able to induce CTE development [14] and to provoke a reduction of cerebral perfusion in thalamus, optical radiation, and other white matter areas [7]. The most frequently observed neuropsychological alterations in subjects with a history of TBI concern information processing speed, executive functions, and associative memory [15, 16]. At later stages, in full-blown CTE, there is also more severe cognitive impairment with attention and language difficulties and memory loss.

The aims of this study were (1) to investigate the possible relationship between the neuropsychological profile of active American football players and their traumatic load; (2) to study if TBI associated with American football activity is associated with a specific cerebral perfusion pattern; (3) and if this perfusion pattern correlates with neuropsychological performances.

## Methods

### Sample

We evaluated cognitive performances and magnetic resonance imaging findings in 20 American football players enrolled according to the following inclusion criteria:

- Having practiced American football for at least 1 year in Italian First Division Championship (Italian Football League — IFL), Second Division and/or under 19 Youth Championship belonging to the FIDAF (Federazione Italiana di American Football), sport affiliated with CONI (Comitato Olimpico Nazionale Italiano);
- Having played American football for at least 2 years and currently playing or having ended the competitive activity within 2 years, as the minimum significant latency for symptoms of CTE is 24 months from the beginning of the cumulative head trauma and not from the end of it [12];
- Male gender;
- Absence of contraindications to the execution of MRI;
- Absence of major neurological or systemic comorbidity.

We divided the football players into 3 groups according to their field position (type 1, 2 or 3 — see below) and into two groups according to their career length (≤ 7 or > 7, i.e., the median of career length observed in our athletes). Field positions were categorized as follows:

1. Line field positions (offensive and defensive linemen). Athletes in these field positions predominantly suffer a high number of hits on the helmet (up to 5 per play and up to 300 in a single game) but on average each single hit is minimally intense. This kind of traumatism may be labeled as “quantitative TBI.” The line positions are those that seem to mostly correlate with the development of neurological sequelae [14, 17];
2. Quarterback, wide receiver, defensive back, special team (except kick returner). Athletes in these field positions suffer few traumatic injuries, but often severe and concussive, both during the game and in practice, because they occur in open field situations, with high speed or where the player cannot see the hitter before the impact (blind side hits);
3. Running back, tight end, linebacker, kick returner. Athletes in these field positions receive frequent and potentially intense hits.

For the neuroimaging study, we recruited a control group of healthy males without TBI history and without major neurological or systemic pathologies, matched by age.

The study was carried out according to the ethical principles of the Helsinki Declaration for medical research. The subjects expressed their written consent to participate in the study.

### Clinical and neuropsychological assessment

We obtained information about sport history (such as field position, years of career, and age), medical history, athletes’ traumatic load and the possible reported symptoms using the TraQ (Trauma Questionnaire) [8]. We measured cognitive performances in players with seven neuropsychological tests to evaluate attention, orientation ability, language, verbal memory, visuospatial and visuo-perceptive ability, executive functions, visual-motor spatial planning, phonemic and semantic verbal fluency, and constructional apraxia:

1. Montreal Cognitive Assessment (MoCa — [18, 19]);
2. Rey-Osterrieth Complex Figure Test [20];
3. Trail Making Test (TMT, [21, 22]) version A and B;
4. F.A.S. Verbal Fluency Task [23];
5. Semantic Verbal Fluency Test [24];
6. Stroop Test — short version [25];
7. Symbol Digit Modalities Test [26].

The details of the neuropsychological tests are provided in the Supplementary information S1.

### MRI acquisition

American football players and controls underwent a MRI evaluation on a 1.5 T Siemens Avanto scanner (Erlangen, Germany). The acquisition comprised (1) a 3D high-resolution magnetization-prepared rapid gradient echo (MPRAGE) T1-weighted image (repetition time (TR) = 1900 ms, echo time (TE) = 3.3 ms, inversion time (TI) = 1100 ms, matrix size = 192 × 256 × 176, resolution = 1 mm3 isotropic); (2) a 3D gradient and spin echo (GRASE) multi-delay pseudocontinuous arterial spin labeling (pCASL) with background suppression sequence (TR = 3500 ms TE = 22.58 ms, labeling duration = 1500 ms, 5 post-labeling delays (PLD) = 700/1200/1700/2200/2700 ms, 12 pairs of tag/control volumes, matrix size = 64 × 64 × 32, resolution = 3.5 × 3.5 × 5 mm3).

### Image processing

The MRI datasets analysis described below was performed by means of FMRIB Software Library toolboxes (FSL, http://www.fmrib.ox.ac.uk/fsl) when not otherwise specified.

#### Structural images

The MPRAGE was skull stripped by means of FSL bet toolbox and the brain tissues were segmented, using the Sienax algorithm [27], in gray matter (GM), WM and cerebrospinal fluid (CSF).

#### pCASL (pseudo-continuous arterial spin labeling) dataset

The tag and control images were preprocessed performing the standard procedures of averaging, realignment and motion correction, using ANT’s software package. Then, the estimation of CBF maps was performed using Oxford_asl followed by their calibration by means of asl calib tools [28]; the required parameters were set as follows: T1 of blood tissue = 1.2 s, T1 of blood = 1.36 s, tagging efficiency = 0.8 accordingly to [29]. Finally, a partial volume correction was performed, and successively the CBF maps were nonlinearly registered, using ANTS, to the MNI standard space, using GM and WM masks derived from the MPRAGE. For each subject, the average perfusion, measured by CBF as ml/100 g/min, was extracted from a total of 62 cortical and subcortical parcels per hemisphere accordingly to the Harvard–Oxford atlas (http://www.cma.mgh.harvard.edu/fsl_atlas.html; http://fsl.fmrib.ox.ac.uk/fsl/fslwiki/Atlases).

### Statistical analysis

The Analysis of Covariance (ANCOVA), covaried by age and education, was used to compare the neuropsychological scores among the three field positions, and between the American football players with a career length longer than 7 years vs those with a career shorter than or equal to 7 years. The post hoc comparisons were performed using the Least Significant Difference (LSD) method. A voxel-wise ANCOVA was performed on normalized CBF maps by means of FSL randomize tool [30] to test perfusion alterations between the American football players and healthy control groups. In this comparison, age was included as covariate of no interest and results were considered statistically significant if the corrected *p*-value (family wise error, FWE) was pFWE ≤ 0.05. Statistically significant differences were anatomically mapped using Harvard–Oxford atlas [http://fsl.fmrib.ox.ac.uk/fsl/fslwiki/Atlases]. The local CBF values, extracted from the Harvard–Oxford parcels, were correlated with NPS scores. This was carried out using partial correlation analysis with age and education as covariates. The resulting *p*-values were corrected for multiple comparison according to the false discovery rate correction [31] using Matlab (MathWorks, Natick, MA, USA, version 2013a). Results with a pFDR ≤ 0.0180 were considered statistically significant.

## Results

### Characteristics of the participants

The demographic characteristics of the American football players are showed in Table 1. The 19 male healthy controls were age-matched with the players, with a median [25th–75th percentile] age of 28.0 [24.8–32.3] years (*p* = 0.235).

**Table 1:** Demographic features of the American Football players. Median [25th–75th percentile] are provided for the continuous variables

American football players	
Number	20
Type 1, 2, 3	5/8/7
Career duration > 7 years (%)	50%
Sex	All males
Age (years)	25.0 [21.6–31.2]
MoCA	23.59 [22.0–25.1]
TMT A (s)	21 [18.5–26.5]
TMT B (s)	53 [44.2–62.0]
TMT B-A (s)	31 [24.0–39.7]
FAS	44 [40.2–51.7]
Stroop (time, s)	11 [9.0–14.4]
Stroop (error #)	0 [0.0–0.0]
Semantic fluency	57 [53.0–63.7]
SDMT	81 [74.0–85.5]

### Neuropsychological assessment

All neuropsychological test performances of the American football players were within normal range (Table 1).

**Fig. 1:**
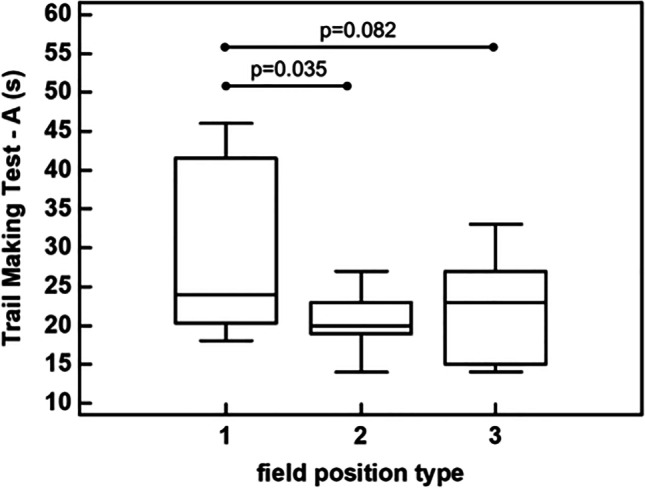
Comparison of the Trial Making Test A performance among field positions (type-1, type-2, type-3)

The athletes with type-1 field position showed significant longer timings during the Trail Making Test—A (24.0 [20.2–41.5] s) compared to the type-2 (20.0 [19.0–23.0] s) (*p* = 0.035), and a trend for longer timing compared to type-3 (23.0 [15.0–27.0] s) (*p* = 0.082) (Fig. 1).

**Fig. 2:**
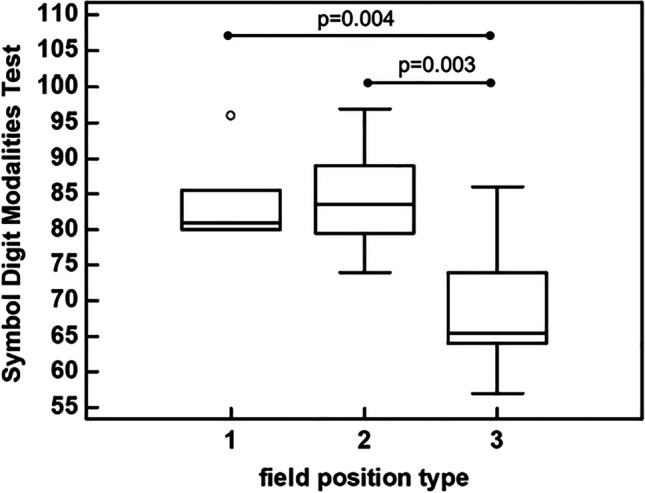
Comparison of the Symbol Digit Modalities Test performance among field positions (type-1, type-2, type-3)

In the Symbol Digit Modalities Test, the athletes with type-3 field position pronounced fewer numbers (65.5 [64.0–74.0]) compared to those in the field position of type-1 (81.0 [80.0–85.5]) (*p* = 0.004), and type-2 (83.5 [79.5–89.0]) (*p* = 0.003) (Fig. 2).

**Fig. 3:**
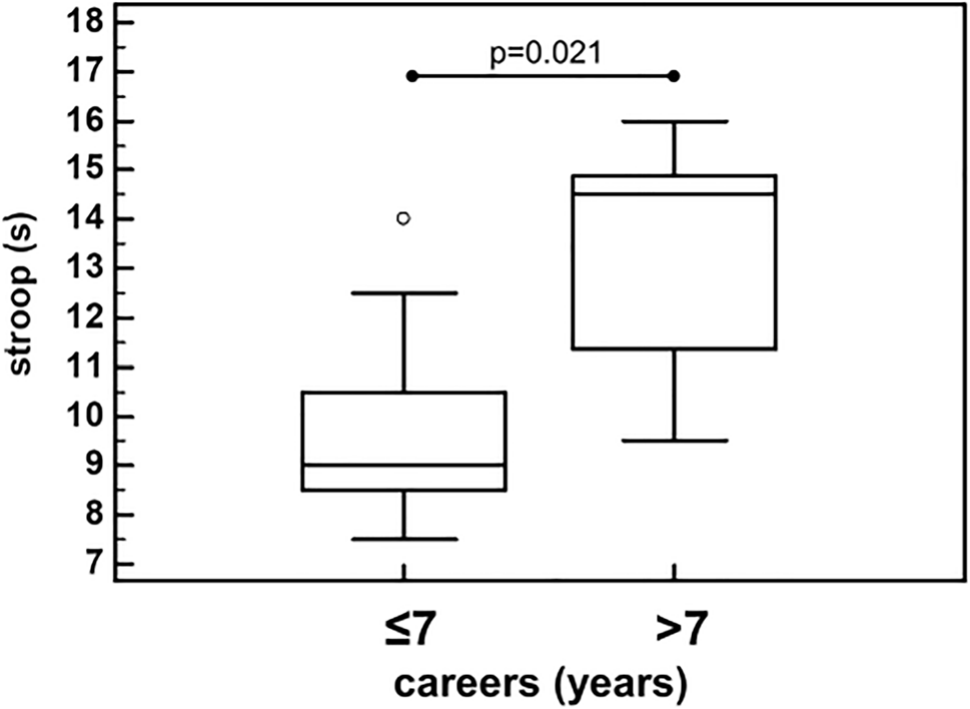
Stroop test performances of athletes with career duration > 7 years or below/equal to 7 years

**Fig. 4:**
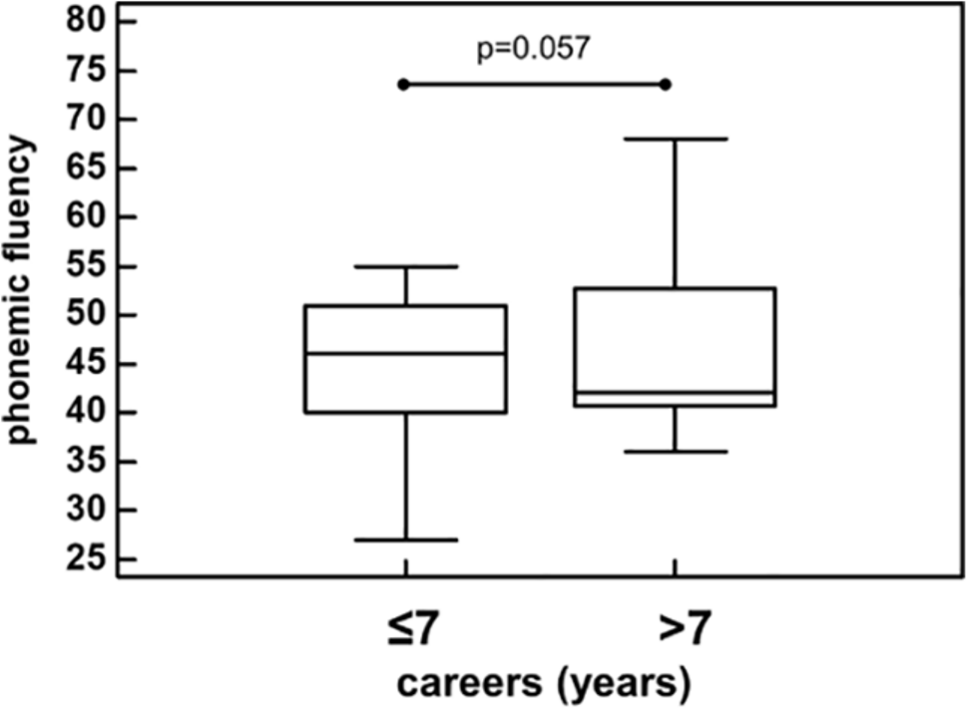
Comparison of phonemic fluency performances of athletes with career duration > 7 years or below/equal to 7 years

The athletes with career longer than 7 years compared to those with career shorter than or equal to 7 years had significantly worse performances of the Stroop (14.5 [11.4–14.9] s vs. 9.0 [8.5–10.5] s, *p* = 0.021) (Fig. 3) test, and a trend for worse phonemic fluency (42.0 [40.7–52.7] vs. 46.0 [40.0–51.0], respectively, *p* = 0.057) (Fig. 4).

No other neuropsychological test performances were significantly different among groups.

### MRI results

**Fig. 5:**
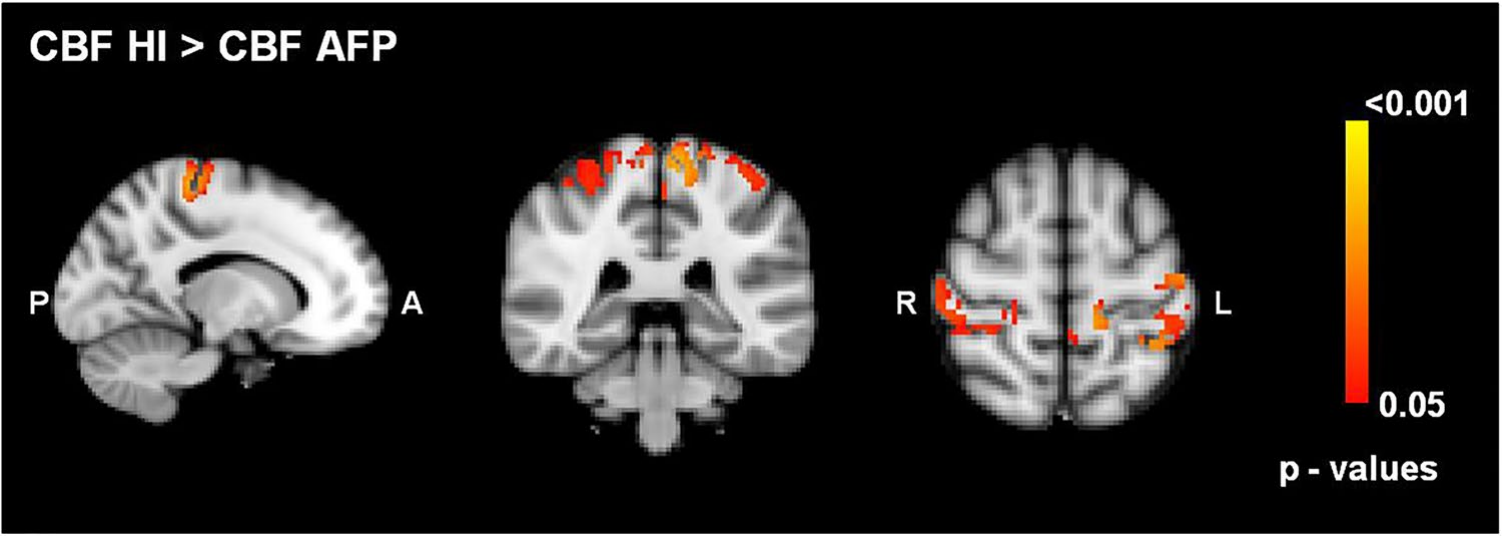
This CBF evaluation, obtained with ASL MRI, shows red colored areas as those characterized by higher CBF in HI (healthy individuals) with respect to AFP (American football players)

The voxel-wise comparison of the CBF maps between AFP and HI revealed a statistically significant (pFWE < 0.05) hypoperfusion in sensory-motor areas, specifically pre- and postcentral gyri (Fig. 5).

The results of partial correlation analyses revealed an association between NPS scores and local CBF values.

A positive correlation (*R* = 0.647, *p* = 0.005) was found between superior parietal lobe CBF values and executive performance of MoCA test scores; a positive correlation was found between CBF and Rey figure test—copy in frontal pole (*R* = 0.573, *p* = 0.016), superior frontal gyrus (0.590, *p* = 0.013), temporal pole (*R* = 0.577, *p* = 0.015), posterior part of inferior temporal gyrus (*R* = 0.665, *p* = 0.004), supplementary motor area (juxtapositional lobule cortex, *R* = 0.625, *p* = 0.007) and orbitofrontal cortex (*R* = 0.671, *p* = 0.003). A negative correlation was found between TMT-B test and local CBF in supramarginal gyrus (*R* =  − 0.574, *p* = 0.016) and in the orbito-frontal cortex (*R* =  − 0.612, *p* = 0.009). Also, TMT B-A tests were negatively correlated (− 0.733 < R ≤  − 0.566 e 0.001 < *p* ≤ 0.018) with local CBF in several brain areas such as precentral gyrus, frontal pole, fronto-orbital cortex and precuneus cortex. Finally, Stroop test score (reading time) was negatively correlated with perfusion data in the middle frontal gyrus (*R* =  − 0.648, *p* = 0.005) and superior parietal lobule (*R* =  − 0.752, *p* < 0.001).

## Discussion

In this study, we found different cognitive performances between American football players stratified according to their field position and career length, statistically significant hypoperfusion in sensory-motor areas, and poorer neuropsychological performances correlated with lower perfusion in specific brain areas in these athletes.

Previous studies frequently failed in the identification of a real traumatic risk of certain sports because they considered all team players as a homogeneous group [9, 14, 32–35]. The introduction of the concept of traumatic load obtained with the TraQ questionnaire allowed us to identify, in the same team, subjects with a specific cognitive traumatic risk, categorizing field positions. We showed differences in cognitive performances in American football players according to their field position and career length. Although the performances of all the players were within normal limits, different field positions were associated to different cognitive performances. In particular, the type-3 and type-1 are associated with worse executive functioning, verbal competence, and visual abilities. Indeed, the type-3 position, the one who receives frequent and potentially intense hits had worse performance of the Symbol Digit Modalities Test compared to the Type-1 position, and even worse compared to the type-2 position that suffers few traumatic injuries. Type-1 field position, the one of offensive and defensive linemen, known to be related to more frequent low intensity TBIs, was found to be associated with poorer performance of the TMT-A. Longer career was associated to poor executive functioning, in particular to a worse Stroop result and a trend for worse phonemic fluency. These observations may induce to hypothesize that frequent minor head trauma, repeated for a long time, is more dangerous for developing at least an impairment of cognitive abilities, which can be the beginning of CTE, characterized by cognitive impairment, mood disorders, behavioral dyscontrol, and focal neurological deficits. Interestingly, these cognitive functions depend on those cerebral areas which have been found to have reduced perfusion at the ASL evaluation. It can be hypothesized that low intensity recurrent TBIs may induce microvascular damage that in turn produces a progressive neuronal loss. From this point of view, the traumatic effect on neurons should be mediated by a vascular functional and/or anatomical dysregulation. Longitudinal studies will be required to identify if the vascular impairment precedes or follows the neuronal loss and the subclinical cognitive decline.

The hypoperfusion found in pre- and postcentral gyri may be interpreted in different ways; more expert players (the ones with a longer career) may have motor neural circuits more trained and more efficacious and consequently with a reduced energy consumption that requires a lower grade of perfusion. Alternatively, we may hypothesize that the summation of recurrent mild trauma is able to induce a slow but progressive neuronal density decrease [2] and consequently, a reduction in energy consumption that in turn may be supported by a lower cerebral perfusion.

The regular use of TraQ might permit to identify specific sportsmen categories at risk of cognitive decline. It seems interesting that American football players in Italy showed, in a previous comparative study, a lower amount of cognitive performances reduction compared to US players [36]. The authors concluded that the possible cause of this difference was the lower amount of contact activity (games and training) in Italy and an older age of starting the American football activity (respectively 9 years in USA and 16.7 in Italy). This is in line with our observation that cognitive impairment is due to a temporal summation of low intensity recurrent traumatic events.

Furthermore, the observation that starting this sport practice at younger age in American football is related to an increased risk of developing cognitive disturbances induces to another consideration: the same traumatic load in terms of frequency and intensity may induce greater damages in a not fully developed brain. This must increase the attention on the juvenile categories of athletes.

Previous studies found that global cerebral perfusion is reduced in sport-associated TBI and especially in the following areas: cingulate cortex, left inferior frontal cortex, thalamus, middle and upper frontal cortex, left pre-central and post-central cortex, left transverse temporal cortex, precuneus, anterior cingulate and cerebellum [4, 6]. Moreover, brain hypoperfusion is implicated in the evolution of damage consequent to TBI, especially associated with edema, contusions and bleeding. This evidence would allow to attribute to MRI ASL technique a predictive and prognostic role of the posttraumatic functional outcome as well as to better define the severity of the TBI itself [37].

A limitation of this study is the small sample size and the current absence of follow-up data.

Future studies should also assess early brain alterations due to mild TBI, like microbleeds, using specific imaging techniques. For example, susceptibility weighted imaging (SWI) MRI is able to identify cerebral microbleeds, iron, and tau protein deposits, of which the role in pathogenesis and development of CTE is demonstrated [17, 38–40]. A second task for future studies would be to perform long follow-up of athletes including neuropsychological and brain perfusion evaluations, and eventual rehabilitative strategies for preventing or avoiding the cognitive worsening.

In conclusion, our study pointed out diminished cognitive functions and the coexisting dysregulation of the cerebral local perfusion in the same cortical areas that resulted impaired. From this perspective, it could be hypothesized that MRI functional impairment and structural damages induced by repetitive, low energy trauma are the two faces of the same medal. The observation of the hypoperfusion of the impaired areas highlights the importance of microcirculation in TBI even if we cannot support, on the basis of these results, that the vascular dysregulation is the mediator between trauma and cerebral hypofunction or assess whether it is one of a series of consequences of the same traumatic situation.

Another relevant information we obtained is the importance to subdivide athletes of the same sport according to specific traumatic load when the effect of TBI of a specific sport is under investigation. Field position, the length of a career, the amount of practices and games, are all relevant elements that influence the traumatic effect of a sport in different ways on each player.

## Supplementary Information

Below is the link to the electronic supplementary material.

Supplementary file1 (DOCX 18 KB)
