# Supplementary material for: Incipient chronic traumatic encephalopathy in active American football players: neuropsychological assessment and brain perfusion measures
Source: Neurol Sci. 2022 Jun 24;43(9):5383–90. doi: 10.1007/s10072-022-06212-7 (PMC9385804; doi:10.1007/s10072-022-06212-7)
Supplement: Supplementary file 1 — Supplementary file1 (DOCX 18 KB) [file 10072_2022_6212_MOESM1_ESM.docx]

**SUPPLEMENTARY MATERIALS**

**S1**

**Neuropsychological tests used for assessment:**

1. **Montreal Cognitive Assessment** (MoCa – [18, 19]: this test evaluates general cognitive functions in different cognitive domains. It is predominantly used as a tool for rapid screening of mild cognitive impairment. The administration time is 10 minutes, the score ranges from 0 to 30 and the cut-off for the identification of pathological mild cognitive impairment is 15.5 using the correction of Santangelo et al. 2014 [19] which appears to be the most useful correction in young subjects. MoCA consists in the evaluation of six domains: 1-Memory: delayed recall test of 5 names after 2 verbal presentations (range 0-5); 2- Visuospatial abilities: clock drawing test (3 points), cube imitation test (1 point) (range 0-4); 3- Executive functions: short version of TMT-B (1 point), phonemic fluency (1 point), two verbal abstraction tests (2 points) (range 0-4); 4- Sustained attention, concentration and working memory: verbal stimulation tapping test (1 point), serial subtraction (3 points), forward and backward digit span (1 point each) (range 0-6); 5- Language: name of animals (3 points), repetition of two syntactically complex sentences (2 points) and the aforementioned phonemic fluency test (1 point) (range 0-6); 6- Spatial and temporal orientation evaluated with questions (range 0-6).
2. **Rey-Osterrieth Complex Figure Test** [20]: this test evaluates visuospatial abilities, visual constructive abilities and visual memory and recently it has also been used for the evaluation of prefrontal lobe executive functions. The subject is asked to copy the figure (for a qualitative assessment it is recommended to provide a succession of 6 pencils of different colours during the test, in our study we used 3 pencils). After 10 minutes, occupied by other non-interfering tests (non-visual spatial nature), it is required to draw the figure by recall. Each element awards 2 points if it is complete and in the right place, 1 point if incomplete or in the wrong place, 0.5 points if incomplete and in the wrong place, 0 points if the element is completely missing. The cut-off of the copy is 28, the delayed reproduction cut-off (10’) is 6.2.
3. **Trail Making Test** (TMT, [21, 22]): this test evaluates how to proceed in visual and visual-motor tasks. It is one of the most frequently used neuropsychological tests due to its simplicity of administration and sensitivity in detecting brain damage. It includes two versions, A and B. In TMT-A, a line is required to be drawn as quickly as possible to connect a series of scattered numbers; this evaluates primarily visuo-perceptive abilities and secondarily attention (cut-off = 93"). In TMT-B, the subject is asked to connect alternatively numbers and letters to evaluate also cognitive flexibility and execution of multiple tasks simultaneously as components of executive functions (cut-off = 282”). The results are expressed in time (seconds) needed to complete the single version of the test, other measurements of the executive functions are the total time needed to complete the two versions and the difference between the two indices (TMT-BA; cutoff = 186").
4. **F.A.S. Verbal Fluency Task** [23]: this test evaluates executive functions, extension and usability of the subject's phonemic access to lexicon in which the patient has to say the largest possible number of words in a limited time interval (one minute) starting with a given letter (F, A, S). Cutoff = 17,35.
5. **Semantic Verbal Fluency Test** [24]: this test evaluates executive functions, extension and usability of the subject's semantic access to lexicon in which the patient has to say the largest possible number of words in a limited time interval (one minute) that belong to a given category (fruits, animals, car brands). Cutoff = 25.
6. **Stroop Test – short version** [25]: this test evaluates executive functions related to selective attention by detecting the tendency to color-word interference. The test consists of three parts and each part consists of three pages: 1. In the first part ("reading" - R), the subject has to read a list of words as quickly as possible and without making mistakes. The examiner has to start timing when the test starts, at the end of the first page, the examiner must turn it quickly, without stopping the time and without interrupting the subject, who can thus continue to read the words on the second sheet. The same applies to the second sheet. At the end of the third page, the examiner blocks the stopwatch and marks both the time and the errors. 2. In the second part (“colour” - C), the subject has to gradually name the colours of the dots shown on the sheet, as quickly as possible but without making any mistakes. After the beginning, the examiner starts timing. At the end of the first page, the examiner must turn it quickly, without stopping the time and without interrupting the subject, who can thus continue to name the colours of the second sheet. At the end of the third page, the examiner blocks the stopwatch and marks both the time and the errors committed by the subject. 3. In the third part (“interference” – I), the subject has to name the colours of coloured words without reading the words themselves, as quick as possible, without making any mistakes. In the end the examiner has to mark both the time and the errors.
7. **Symbol Digit Modalities Test** [26]: this test evaluates the speed with which a piece of information is analyzed and added to data usable in other cognitive processes. Both the sustained attention (functional component of short-term memory that includes vigilance and working memory) and selective attention (ability to avoid distractions from other stimuli), as well as visual-spatial abilities are implicated in this process. The test consists in reading numbers corresponding to symbols using, when necessary, the legend shown at the top of the sheet. The normal cut-off is 34.2 words read in 90". In this study, we administered the oral version and we continued the test beyond 90", until all 110 symbols were exhausted. We also reported time and number of errors for an additional assessment of the sustained attention.
